# Prevalence of pathogenic variants in DNA damage response and repair genes in patients undergoing cancer risk assessment and reporting a personal history of early-onset renal cancer

**DOI:** 10.1038/s41598-020-70449-5

**Published:** 2020-08-11

**Authors:** Tiffiney R. Hartman, Elena V. Demidova, Randy W. Lesh, Lily Hoang, Marcy Richardson, Andrea Forman, Lisa Kessler, Virginia Speare, Erica A. Golemis, Michael J. Hall, Mary B. Daly, Sanjeevani Arora

**Affiliations:** 1 Arcadia University, Glenside, PA USA; 2 Cancer Biology Program, Fox Chase Cancer Center, Philadelphia, PA USA; 3 Cancer Prevention and Control Program, Fox Chase Cancer Center, 333 Cottman Avenue, Philadelphia, PA 19111-2497 USA; 4 Molecular Therapeutics Program, Fox Chase Cancer Center, Philadelphia, PA USA; 5 Kazan Federal University, 420000 Kazan, Russian Federation; 6 Geisinger Commonwealth School of Medicine, Scranton, PA USA; 7 Ambry Genetics, Konica Minolta, Aliso Viejo, CA USA; 8 Department of Clinical Genetics, Fox Chase Cancer Center, Philadelphia, PA USA

**Keywords:** Cancer genetics, Cancer prevention, Cancer screening, Urological cancer, Kidney diseases, Cancer genetics, Cancer screening, Urological cancer

## Abstract

Pathogenic variants (PVs) in multiple genes are known to increase the risk of early-onset renal cancer (eoRC). However, many eoRC patients lack PVs in RC-specific genes; thus, their genetic risk remains undefined. Here, we determine if PVs in DNA damage response and repair (DDRR) genes are enriched in eoRC patients undergoing cancer risk assessment. Retrospective review of de-identified results from 844 eoRC patients, undergoing testing with a multi-gene panel, for a variety of indications, by Ambry Genetics. PVs in cancer-risk genes were identified in 12.8% of patients—with 3.7% in RC-specific, and 8.55% in DDRR genes. DDRR gene PVs were most commonly identified in *CHEK2*, *BRCA1, BRCA2,* and *ATM*. Among the 2.1% of patients with a *BRCA1 *or* BRCA2* PV, < 50% reported a personal history of hereditary breast or ovarian-associated cancer. No association between age of RC diagnosis and prevalence of PVs in RC-specific or DDRR genes was observed. Additionally, 57.9% patients reported at least one additional cancer; breast cancer being the most common (40.1% of females, 2.5% of males). Multi-gene testing including DDRR genes may provide a more comprehensive risk assessment in eoRC patients. Further validation is needed to characterize the association with eoRC.

## Introduction

Renal cancer (RC) often develops with no signs or symptoms and is referred to as the “silent disease”^1^. While factors including smoking, environment, obesity, and race have been linked to increased risk of RC, inherited factors are the most well-validated source of increased risk^2–4^. Hereditary RC syndromes, typically associated with early-onset disease and a clinically significant family history of cancer, result from germline pathogenic variants (PV) in high-penetrance ‘RC-specific’ genes including *VHL, MET, FLCN, TSC1, TSC2, FH, SDH, PTEN* and *BAP1*^5–7^. A previous report of an early-onset RC (eoRC) cohort screened with an RC-specific panel found 6.1% of individuals had a PV in an RC-specific gene^7^. However, for most eoRC patients a PV in an RC-specific gene is not identified, leaving many eoRC genetically undefined. Thus, there is a need to identify additional genes related to eoRC risk. Currently, there are no National Comprehensive Cancer Network (NCCN) guidelines for detection, prevention, or risk reduction in individuals who present with an eoRC but lack a PV in a defined RC-specific gene^8^.

DNA damage response and repair genes (DDRR) play an important role in maintaining genome integrity, and when mutated in the germline can increase cancer risk for several types of cancers, including breast, colorectal, ovarian, and others^9^. Although PVs in DDRR genes are associated with increased risk of a variety of cancer types, they are not typically considered risk factors for RC. However, germline PVs in some DDRR genes have been observed in RC, including PVs in the DNA mismatch repair (Lynch syndrome) genes *MSH2* and *MLH1* in renal urothelial carcinoma, and PVs in *CHEK2* in advanced renal cell carcinoma^10–21^. To address the hypothesis that PVs in additional DDRR genes may contribute to the missing heritability of eoRC, we analyzed germline sequencing data from a cohort of 844 individuals with RC.

## Materials and methods

### Ambry Genetics eoRC study cohort, and variant determination

De-identified data were requested from RC patients that were tested by Ambry Genetics (Konica Minolta, Aliso Viejo, California) using germline cancer testing panels. Ambry samples were selected for patients with RC, and de-identified data was obtained for all RC patients tested with multi-gene cancer panels (n = 844, ≤ 60 years at diagnosis, specimens collected between July 2012–December 2016). All genetic test results from germline testing of individuals diagnosed with RC at ≤ 60 during this time period were used in this study.

There is currently no standard definition specifying the age when RC is considered early-onset. Different models have been used to determine a specific age as a trigger for germline testing in patients with RC who lack family history of RC, including ages < 46^22^ or < 40^7^ years. For this study, we selected individuals 60 years or younger as the cut-off for our cohort, which is substantially below the median age of RC diagnosis of 64 years in the general population as reported in SEER^22,23^, but considerably older than other suggested cut-offs. We did so because the main hypothesis of the study was that PVs in DDRR genes might be responsible for increased risk of RC. Variants in multiple DDRR genes are associated with early-onset colorectal cancer^24,25^, which typically manifests in patients at 50 years or younger. We considered that PVs in DDRR genes were most likely to impact repair of DNA damage induced during cell replication, leading to genetic instability and cancer; given renal cells turn over much less frequently than colon cells, we hypothesized that it may take longer for cancers associated with PVs in DDRR genes to manifest in RC, causing us to select a cut-off of ≤ 60 years old for assessment.

De-identified data included family history of cancer, genetic test results, personal history of cancers (apart from RC), presence of multifocal tumors, and RC-subtype/stage. The RC patients had been tested with CancerNext versions 1–4, and CancerNext-Expanded versions 1 and 2 (Table S1). De-identified patient information was analyzed for genetic test results, and personal and family medical histories. Classification of variants by Ambry Genetics is based on ACMG recommendations for standards for interpretation and reporting of sequence variations. These variants are also regularly deposited in ClinVar by Ambry Genetics. Variant classification was updated through March 31, 2018 for all data. Gene variants were classified as pathogenic variant (PV—see below for criteria), variant of uncertain significance (VUS) or inconclusive, or negative/indeterminate. Ambry Genetics follows strict criteria when classifying variants as PV, Variant Likely Pathogenic (VLP), VUS, Variant Likely Benign (VLB) and Benign (for details see https://www.ambrygen.com/clinician/our-scientific-excellence/variant-classification). Variants reported as PV and VLPs were grouped as PVs. All test results were used for this study. The analysis of VUS, which currently lack clinical significance, was beyond the scope of this study. Given updating of test panels by Ambry Genetics, not all patients were tested for all genes. Individuals were provided different versions of the panel over the course of the study (see below and also see Table S1).

Any de-identified personal or family history information including sex, ethnicity/race, age of cancer diagnosis, tumor histology, history of additional personal cancer, and history of family cancer and types was reported first as summarized data and later as de-identified individual case reports. For analysis comparing outcomes for RC-specific genes versus genes not typically associated with RC, we focused our statistical comparison on only those individuals who had CancerNext Expanded panel version 2 testing which analyzes all 49 genes including the RC-specific genes. 491 individuals who had the CancerNext Expanded version 2 test were used for this statistical comparison. For additional statistical test comparisons that analyzed the correlations between specific genes and categories such as tumor pathology or age, any individual who had been tested for that specific gene was included.

The Western IRB issued a regulatory opinion that the Genomic Data Sharing Policy for Ambry Genetics does not involve human subjects based on 45 CFR46.102(f) and associated guidance, thus the requirement to obtain written patient informed consent was waived. A Data Use Agreement, and Materials Transfer Agreement was established between Ambry Genetics and Fox Chase Cancer Center. The FCCC Institutional Review Board (IRB) provided study oversight and approval (protocol number 14831). Ambry Genetics provided de-identified patient medical and family history (where available), and genetic results for the patients. All methods were performed in accordance with the relevant guidelines and regulation of the approved study.

### Genetic analysis with Ambry CancerNext and CancerNext Expanded panels

Individuals were provided different versions of the panel by their healthcare provider (see Table S1). The number of genes in the panels ranged from the smallest CancerNext panel Version 1 which include 22 genes (*APC, ATM, BARD1, BRIP1, BMPR1A, CDH1, CHEK2, EPCAM, MLH1, MRE11A, MSH2, MSH6, MUTYH, NBN, PALB2, PMS2, PTEN, RAD50, RAD51C, SMAD4, STK11, TP53*) to the largest CancerNext Expanded Version 2 panel, which contained 49 genes (*APC, ****ATM, ****BAP1,****BARD1, BRCA1, BRCA2, BRIP1,**** BMPR1A, CDH1, CDK4, CDKN2A, ****CHEK2,**** EPCAM, FH, FLCN, GREM1, MAX, MEN1, MET, MITF,****MLH1****, ****MRE11A, MSH2, MSH6, MUTYH, NBN,**** NF1,****PALB2, PMS2, POLD1, POLE,****PTEN,****RAD50, RAD51C, RAD51D,****RET, SDHA, SDHAF2, SDHB, SDHC, SDHD, SMAD4, SMARCA4, STK11, TMEM127, TP53, TSC1, TSC2, VHL*). The DDRRs identified in germline testing of this cohort are bolded^26^.

Ambry Genetics sequenced genomic DNA that was obtained from patient blood or saliva samples. DNA was evaluated by next generation sequencing (NGS) of all coding sequences, and ± 5 bases into the 5′ and 3′ ends of flanking introns and untranslated regions. In addition, sequencing of the promoter region was performed for the following genes: *PTEN* (c.− 1,300 to c.− 745), *MLH1* (c.− 337 to c.− 194), and *MSH2* (c.− 318 to c.− 65). Additional Sanger sequencing was performed for any regions missing or with insufficient depth of coverage for reliable heterozygous variant detection, and on potentially homozygous variants, variants in regions with complicated pseudogene interference, and when variant calls did not meet allele frequency quality thresholds. Additional details on specific testing methods are available at https://www.ambrygen.com/clinician/genetic-testing/28/oncology/cancernext-expanded.

### Control population in ExAc and gnomAD

To compare the frequency of DDRR gene PVs found in the study to that in the general population, our results were compared to the Exome Aggregation Consortium (ExAc) dataset of largely unrelated ~ 60,000 whole exome sequencing results, and to the Genome Aggregation database (gnomAD) dataset consisting of ~ 125,000 exomes and ~ 15,000 genomes^27,28^. These datasets are the most commonly used genomic data at the population-level.

### ClinVar analysis

ClinVar (https://www.ncbi.nlm.nih.gov/clinvar/), a database of medically relevant gene variants, was used to investigate all PVs in this study (retrieved on February 4, 2020). PVs that were not reported in ClinVar were noted as ‘*not reported’*. Associated conditions for each PV were categorized into hereditary cancer predisposing syndrome(s), condition(s) related to renal cancer, and any other condition(s). To further elucidate any PVs related to renal cancer, the search term “renal cancer” was queried, and the results were noted as “*associated with ClinVar search term ‘Renal Cancer.*’”.

### Statistical analysis

To identify potential correlations between PVs and characteristics such as tumor pathology, additional primary tumor type, and age of diagnosis, genes were combined into pathways/groups of interest, histology’s were grouped, and cancer types were grouped. Each individual was categorized as having a variant in one of the genes within the group or no variant in the group. Gene categories were used for comparison of RC diagnosis with a DDRR or an RC-specific gene.

We also tested the hypothesis that different gene groups are associated with age at RC diagnosis. We used the median age of RC diagnosis in the study cohort (48 years), and studied PVs in patients < 48 years or ≥ 48 years of age. To test the association between the presence of PVs, and age of RC diagnosis, two-sided Fisher’s exact tests were used, and p-values ≤ 0.05 were considered significant. Odds ratios (OR) were calculated to determine the odds that an outcome had occurred given a particular variant, compared to the odds of the outcome occurring in the absence of that variant in the population tested. Finally, we queried the SEER database for patients under 60 years old with RC to compare the distribution of their clinical characteristics (where available) to those in our study cohort^22^.

Due to the evolving nature of the panels during the course of this study, each version included a different total number of genes, and analysis of each gene is based on the number of individuals whose test included that gene (Table S1). Only data from 491 individuals was considered for comparison of individuals with RC-specific genes compared to those with variants in genes not typically associated with RC, as the other individuals did not have all 49 genes analyzed. For statistical comparisons analyzing correlations between specific genes with various characteristics, all individuals who had been tested for that specific gene were included.

To identify potential correlations between PVs and characteristics such as tumor pathology, additional primary tumor type, and age of diagnosis, RC-specific genes, other cancer-associated genes, and DDRR genes were combined into groups, and histologies were grouped. The categories for comparison of PVs and patient characteristics are as follows:

1. Known RC genes (*BAP1, FH, FLCN, MEN1, MET, MITF, PTEN, SDHA, SDHAF2, SDHB, SDHC, SDHD, TSC1, TSC2,* and *VHL* ) versus genes not typically associated with RC (*APC, ATM, BARD1, BRCA1, BRCA2, BRIP1, BMPR1A, CDH1, CDK4, CDKN2A, CHEK2, EPCAM, GREM1, MAX, MLH1, MRE11A, MSH2, MSH6, MUTYH, NBN, NF1, PALB2, PMS2, POLD1, POLE, RAD50, RAD51C, RAD51D, RET, SMAD4, SMARCA4, STK11, TEMEM127, TP53*) versus DDRR genes alone (*ATM, BARD1, BRCA1, BRCA2, BRIP1, CHEK2, MLH1, MRE11A, MSH2, MSH6, MUTYH, NBN, PALB2, PMS2, POLD1, POLE, RAD50, RAD51C, RAD51D*)*.*
2. Histology categories combined from the original categories: (1) Chromophobe, (2) Papillary renal, (3) Clear cell, (4) Wilms, (5) Renal cell (likely clear cell but cannot be confirmed), (6) Unknown, (7) Mixed papillary [clear cell papillary type, papillary renal/chromophobe renal, and sarcomatoid/papillary/clear cell]^29^, (8) Mixed chromophobe [chromophobe/oncocytoma, chromophobe/renal cell, clear cell/chromophobe, and clear cell/oncocytoma/chromophobe]^30^, (9) Oncocytoma, (10) Mixed oncocytoma [clear cell/oncocytoma, oncocytoma/collecting duct, and renal cell/oncocytoma] ^31^, and (11) Others [included clear cell/sarcomatoid, collecting duct, mixed epithelial and stromal, mucinous tubular and spindle cell, multilocular cystic renal, neuroendocrine, renal cell/Wilms, renal cortical, sarcomatoid, transitional, urothelial and urothelial transitional]. Transitional, urothelial, urothelial and papillary transitional cases were not included in the analysis for counts of pathogenic variants. Renal oncocytomas, mixed epithelial and stromal tumors are considered benign tumors, and were not included in the analysis for counts of pathogenic variants ^32,33^.

### Study approval

The Western IRB issued a regulatory opinion that the Genomic Data Sharing Policy for Ambry Genetics does not involve human subjects based on 45 CFR46.102(f) and associated guidance, thus the requirement to obtain written patient informed consent was waived. A Data Use Agreement, and Materials Transfer Agreement was established between Ambry Genetics and Fox Chase Cancer Center. The FCCC Institutional Review Board (IRB) provided study oversight and approval (protocol number 14831). Ambry Genetics provided de-identified results for the study. All methods were performed in accordance with the relevant guidelines and regulation of the approved study.

## Results

### Patient characteristics

**Figure 1:**
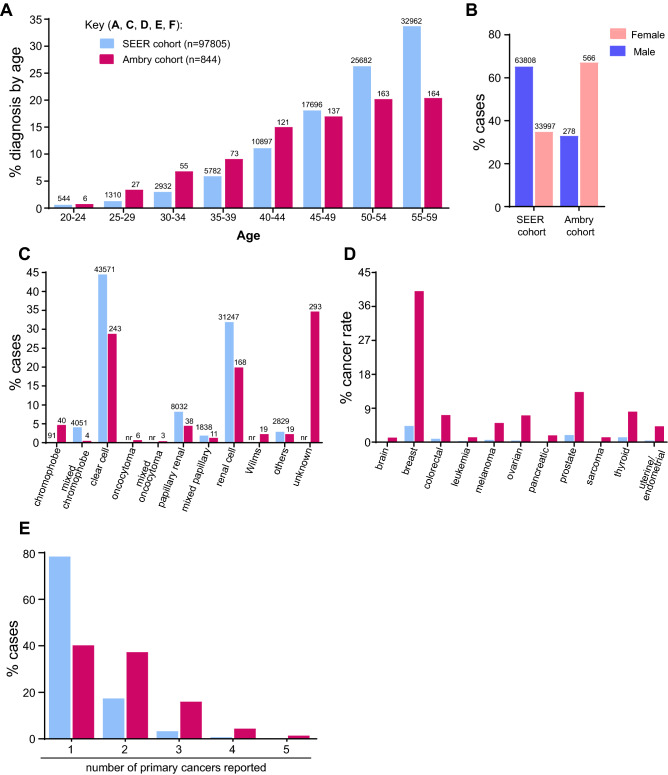
Patient characteristics. (**A**) Age range of individuals diagnosed with RC ≤ 60 years in SEER cohort compared to the study cohort (n = 746; of the remaining individuals in the study, 26 were diagnosed < 19 years, 33 were diagnosed at 60 years, and 39 were excluded from the calculations as their age was reported as a wide range of years). (**B**) Percentage of males and females diagnosed with RC ≤ 60 years in SEER compared to the study cohort (n = 844). (**C**) The percentages of reported RC histology up to age 60 years in the SEER data (n = 97,805) compared to the study cohort (n = 844); not all histological subtypes reported in SEER were reported in the study cohort**. **(**D**) The percentage of cancer incidence (at ≤ 60 years) in the general SEER population versus the study cohort. The SEER data reflect individuals reporting the indicated cancer type, not individuals with RC in addition to the indicated cancer type. (**E**) Percentage of different primary cancers reported (≤ 60 years) in SEER (n = 97,795) versus the study cohort (n = 844). Less than 0.4% not reported for figure clarity.

We first benchmarked the eoRC study cohort to the reported incidence of RC in SEER data for the general US population to provide context. In the study cohort, 40% of cases were between 50–59 years of age, and median age of diagnosis was 48 years. As expected, a higher percentage of RC cases were diagnosed between 20–44 years of age as compared to patients ≤ 60 diagnosed with RC in the general US population (SEER) (35%, versus 21.9%) (Fig. 1A). The study cohort was 67.1% female and 32.9% male (Fig. 1B, Table 1), versus 34.8% female and 65.2% male for the general US population prevalence of RC diagnosed ≤ 60 (Fig. 1B). Race/ethnicities in study cohort were 65.6% Caucasian, 5.8% African American/Black, 5.3% Ashkenazi Jewish, 7.6% Hispanic, 0.5% other, and 5.5% unknown (Table 1).

**Table 1:** Demographics and clinical characteristics of RC patients in the Ambry Genetics study cohort.

Characteristic	Number of patients in Ambry study cohort (%)	Rate in general population from birth to age 60 (SEER)
**Sex**		
Male	278 (32.9%)	65.2% of renal cancers
Female	566 (67.1%)	34.8% of renal cancers
**Ethnicity**		
African American	49 (5.8%)	13.4%
Ashkenazi Jewish	45 (5.3%)	nr
Asian	18 (2.1%)	nr
Caucasian	554 (65.6%)	79.9%
Hispanic	64 (7.6%)	nr
Middle Eastern	4 (0.5%)	nr
Mixed Ethnicity	58 (6.9%)	nr
Native American	2 (0.2%)	nr
Other	4 (0.5%)	5.9%
Unknown	46 (5.5%)	0.7%
Median age of testing	53 years	
**Histology**		
Chromophobe	40 (4.7%)	0.1%
Mixed chromophobe	4 (0.5%)	4.1%
Clear cell	243 (28.8%)	44.5%
Oncocytoma	6 (0.7%)	0
Mixed oncocytoma	3 (0.4%)	0
Papillary renal	38 (4.5%)	8.2%
Mixed papillary	11 (1.3%)	1.9%
Renal cell	168 (19.9%)	31.9%
Wilms	19 (2.3%)	0
Others	19 (2.3%)	6.4%
Unknown	293 (34.7%)	0
**Personal cancer history**		
Renal cancer only	355 (42.1%)	
Renal cancer plus additional cancer type	489 (57.9%)	
**Family history of cancer**		
Yes	784 (92.9%)	
No	9 (1.1%)	
Not reported/unknown	51 (6%)	
Family history of renal cancer*		
Yes	196 (24.7%)	
No	597 (75.3%)	
Total	844	

Demographics and clinical characteristics of the RC cases in the study cohort were compared to those of RC (from birth to age 60) in the SEER data. Personal and family history of cancer were reported for the cases in the study cohort.

*For family history of renal cancers, numbers include only those who reported on cancer history (n = 793). *nr *not reported. SEER data included 149 types of renal cancer histologies, not all were represented in dataset; “other” based on other category from Ambry cohort. Family histories as self-reported on the intake form/medical records and have not been validated.

The tumor pathologies reported varied (Fig. 1C and Table 1). Clear cell constitutes 44.5% of all RCs in SEER, and was the most commonly reported histology in the eoRC cohort (243/844 = 28.8%). Renal cell (not defined, but likely to predominantly reflect clear cell) was also common (168/844 = 19.9%, Fig. 1C and Table 1). Papillary and chromophobe histology were each identified in ~ 4–5% of the individuals (38/844 = 4.5% and 40/844 = 4.7%, respectively). Other histologies were identified rarely, but included Wilms tumor (19/844 = 2.3%) and oncocytoma (6/844 = 0.7%). For 34.7% of patients, the RC subtype was unknown.

### High incidence of other cancers in study cohort

57.9% (n = 489/844) of the cases in the study cohort reported at least one additional primary cancer (Fig. 1D, Table 1, Table S2). Each of the primary cancer types is also represented at a higher level in the study cohort than in the general US population as reported by the SEER database (Fig. 1D). For female-specific cancers, 40.1% of females (227/566) also had breast cancer, in comparison to the 4.3% breast cancer rate in women ≤ 60 in the general population (SEER) (Fig. 1D and Table S2). The rate of additional primary cancer in the study cohort (57.9%) is much higher than the rate of each cancer type observed in SEER cases with eoRC (21.6%) (Fig. 1E). Finally, 784 patients out of 844 reported a family history of cancer, and of these 784 patients, 196 (24.7%) specifically reported at least one family member with RC (Table 1).

### Multi-gene cancer panel testing identifies PVs in DDRR genes in the study cohort

**Figure 2:**
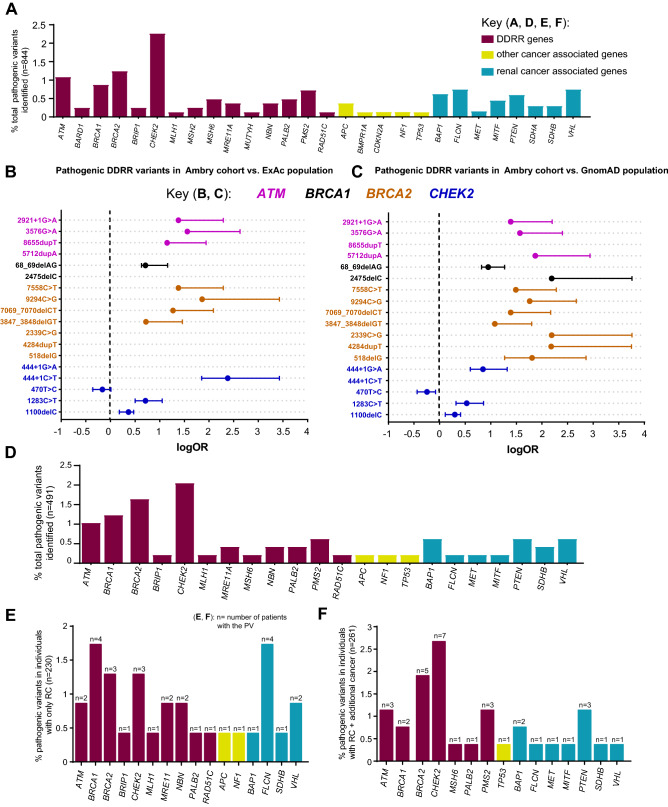
Enrichment of PVs in DDRR genes. (**A**) Cases with germline PVs in the entire cohort (n = 844). (**A**) Red bars; DDRR genes, yellow bars; other-cancer associated genes; blue bars; RC genes. *APC* variants identified in this study were all the moderate risk c.3920T>A, p.I1307K variant, and 5 of the 19 *CHEK2* variants were the moderate risk c.470T>C, p.I157T variant. *The individual with *MUTYH* was a compound heterozygote with two PVs. The data is presented as percent rather than ‘n’ due to the fact that not all 49 genes were tested for all patients in the full study cohort of 844 individuals. The percent adjusts for the number of individuals that were tested for each gene. The ‘n’ values are listed in Supplemental Table 3. (**B**,**C**) Odds of finding PVs in *ATM* (pink circle), *BRCA1* (black circle), *BRCA2* (orange circle)*,* and *CHEK2* (blue circle) from study cohort versus control population, ExAc (**B**) and gnomAD (**C**). Data is presented as log10 odds ratio (OR), and log10 confidence intervals. Dotted black line; association with outcome i.e. OR > 0 is enrichment in study cohort, OR = 0 no difference in cohorts. PVs not found in gnomAD or ExAc are indicated by the absence of any data or PVs not listed from Supplemental Table 4 were not found in the control population. Note: Computation of proportion or burden of individuals with all PVs in a specific gene(s) in the control population cannot be accurately performed as all PVs have not been defined, and while there is some agreement on which variants in a specific gene(s) are currently considered PVs, this is not true for all variants in that gene (as referenced in ClinVar, https://www.ncbi.nlm.nih.gov/clinvar/). (**D**) Cases with germline PVs in the cohort tested for all genes in the study (n = 491, 49 genes). The total ‘n’ is listed in Supplemental Table 6. (**E**) Individuals with germline PVs who were diagnosed with only RC (n = 230/491). The total ‘n’ is shown above the bar as PV per gene. (**F**) Individuals with germline PVs who were diagnosed with RC plus at least one additional primary cancer type (n = 61/491). The total ‘n’ shown above the bar as PV per gene. The color scheme as in (**A**). In (**D**–**F**), to remain consistent between graphs, the data is presented as percent rather than ‘n’ even though all 49 genes were tested for all individuals represented in these graphs.

The most common gene with PVs identified in the eoRC patients was the DDRR gene *CHEK2* (19/844, 2.25%, Fig. 2A, Table S3 and S4), consistent with a recent report by Carlo et al.^16^ Of patients with *CHEK2* PVs, 47.3% (n = 9/19) had a common, highly damaging variant (c.1100delC, p.Thr367Met*fs*) that is known to be associated with an increased risk for breast, prostate, colorectal and thyroid cancers (Table S4)^34–37^.

After *CHEK2*, PVs were most frequently observed in the DDRR genes *BRCA2* (10/815, 1.23%), *ATM* (9/844, 1.07%) and *BRCA1* (7/815, 0.86%) (Table S3). We compared the overall frequency of PVs in *CHEK2, BRCA1, BRCA2,* and *ATM* to the control population in ExAc and gnomAD, representing individuals sequenced for disease-specific and population genetic studies^27,28^. Overall, PVs in each of these genes were more common in the study cohort versus the control populations (Fig. 2B,C, Table S5A). An outlier was the moderate risk *CHEK2* c.470T>C p. I157T PV^38^ identified in 5 individuals in the study cohort, which was higher in the controls (gnomAD- OR, 0.60; 95% CI, 0.234–1.433; ExAc- OR, 0.72; 95% CI, 0.282–1.74). We compared the prevalence of all PVs in DDRR genes presented in Table S4, from 844 cases, to controls from gnomAD^23^. We found ~ 4.8-fold enrichment of PVs in DDRR genes in the study cohort versus the controls in gnomAD (8.4% vs. 1.8% respectively, Table S5B; each DDRR gene was corrected for number of patients assessed).

Cancer risk with *MUTYH* (DDRR gene) has only been defined for individuals with homozygous or compound heterozygous PVs, but not for heterozygous carriers^39^. We identified 17/844 individuals with *MUTYH* PVs, out of which 16/17 were heterozygous carriers and only 1/17 was compound heterozygous. Only the individual with compound heterozygous *MUTYH* PVs was counted in the full study cohort (n = 844, Table S3 and Fig. 2A). Similar to *MUTYH*, cancer risk from the *FH* (RC-specific gene) c.1431_1433dupAAA, p.K477DUP variant is currently considered to be pathogenic only in the compound heterozygous or homozygous state^40^. We identified 2 RC patients who were heterozygous carriers of this specific *FH* variant (Tables S3 and S4).

The overall gene variation rate in the full study cohort (n = 844) is presented in Table S3. The full study cohort was not tested for all 49 genes. The largest panel was tested in the sub-cohort of 491 cases, and consisted of 49 genes, which included 15 RC-specific genes, and 34 other-cancer associated genes including 19 DDRR genes (Table S1). Here, 12.8% (63/491) of cases had PVs. PVs were identified in one or more of the 16 genes not typically associated with RC in 9.16% cases (n = 45/491, Table S6), versus 3.7% (n = 18/491) with a PV in RC-specific genes (Fig. 2D, Table S6). Of the 16 genes not typically associated with RC, 12 were in DDRR genes (8.55%, n = 42/491 or 66.7%, n = 42/63). Among the 491 patients, 2 patients were found to have PVs in two genes. One patient had PVs in two DDRR genes (*BRCA1* and *MUTYH het*), and the other patient in a RC-specific gene and a DDRR gene (*SDHB* and *MUTYH het*) (Table S4). There was no *MUTYH* or *FH* compound heterozygous or homozygous PV in the sub-cohort of 491 cases.

### DDRR genes PVs are similarly enriched in patients diagnosed with eoRC alone, or with eoRC and other cancers

Individuals who were tested for all 49 genes (n = 491) could be further separated into two sub-cohorts: those with eoRC as their only diagnosis (n = 230/491, 46.8%), and those with eoRC and one or more additional types of cancer (n = 261/491, 53.2%). To test the hypothesis that DDRR gene PVs might be associated with eoRC, we first analyzed PVs in eoRC cases with no additional primary cancer diagnosis. Among the 230 patients who only presented with eoRC, PVs were identified in 13% of cases (n = 30/230, Fig. 2E), which is approximately twice the reported frequency of PVs in RC-specific genes^7^. Among this 13%, 8.7% (n = 20/230) of PVs were in one of 10 DDRR genes (*ATM, BRCA1, BRCA2, BRIP1, CHEK2, MLH1, MRE11A, NBN, PALB2, RAD51C*), 3.5% (n = 8/230) were in one of 4 RC-specific genes (*BAP1, FLCN, SDHB, VHL*), and the remaining cases bore PVs in non-DDRR genes associated with cancers other than RC (Fig. 2E).

Next, we performed similar analysis as described above for patients who presented with eoRC plus one or more additional cancers. Among the 261 patients who presented with eoRC and at least one additional cancer, PVs were identified in 12.7% cases (33/261, Fig. 2F). Among these 12.7% of cases, PVs in other-cancer associated genes, including DDRR genes, were found in 8.8% of cases (n = 23/261), versus 3.8% (n = 10/261) of cases with PVs in RC-specific genes. This population was also enriched for PVs in 7 DDRR genes (8.4%, n = 22/261, *ATM, BRCA1, BRCA2, CHEK2, MSH6, PALB2, PMS2*), versus PVs in 7 RC-specific genes (*BAP1, FLCN, MET, MITF, PTEN, SDHB, VHL*).

Overall, these data suggest that DDRR gene PVs are enriched similarly in individuals diagnosed with eoRC alone or eoRC plus at least one additional primary cancer, but that the frequency of PVs in DDRR genes, in either group, exceeded that in the control populations tested (gnomAD/ExAc) (Fig. 2, Table S5A). The specific PVs identified were similar in frequency to those identified in the full patient cohort (n = 844), with *CHEK2* the most represented DDRR genes (Fig. 2). To gain additional insight into the prevalence of these PVs in cancer patients, we surveyed ClinVar (https://www.ncbi.nlm.nih.gov/clinvar/), and found that multiple PVs from this study (Table S4) have been reported in hereditary cancer predisposing syndromes (HCPS, summarized in Table S7). HCPS reflects a pattern of cancers in a family characterized by earlier onset, with individuals not necessarily having the same tumor and/or having more than one primary tumor, and having tumors that are more likely to be multicentric.

### RC patients with *BRCA1* or *BRCA2* PVs

Notably, 1.2% (10/815) of the eoRC cases had a *BRCA2* PV, and 0.9% (7/815) RC cases had a *BRCA1* PV (Table 2, Table S3). This included 1.7% (n = 6/355, Table 2) of the cases who presented with only eoRC. Interestingly, despite the fact that the cohort was 67.1% female, 47.1% (8/17) of the detected *BRCA1* and *BRCA2* PVs were in males (Table 2). Of the 17 RC cases with a *BRCA1 or BRCA2* PV, 8 (47.1%, 8/17) had an additional cancer associated with hereditary breast and ovarian cancer (HBOC) syndrome (breast, ovarian, prostate, pancreatic or melanoma), 3 had an additional non-HBOC cancer (17.6%, 3/17), and 6 presented with only eoRC (35.3%, 6/17) (Table 2). Family history was reported for 16 cases, and of those, 14/16 (87.5%) indicated that at least one family member had an HBOC-associated cancer. Of those with a *BRCA2* PV, 7/10 (70%) reported that at least one family member had RC (Table 2).

**Table 2:** Personal and family history of *BRCA1* or *BRCA2* positive patients in Ambry Genetics RC study cohort.

Gene	Pathogenic variant	Gender	Age—RC diagnosis	Personal cancer history	Age—HBOC syndrome cancer	Family history of cancer
*BRCA1*	c.68_69DELAG p.E23Vfs*17	M**	51	Renal	–	Breast Melanoma Gastric
*BRCA1*	c.2475DELC p.D825Efs*21	F	55	Renal	–	Breast Ovarian
*BRCA1*	c.5207T>C p.V1736A	F	45	Renal	–	Breast Ovarian Gastric Lung
*BRCA1* + *MSH6*	c.68_69DUPAG p.E23Efs*9	M	56	Renal Prostate CRC	46	Ovarian Brain Uterine
*BRCA1*	c.68_69DELAG p.E23Vfs*17	F	50	Renal Ovarian Cervical	63	Not provided
*BRCA1*	c.4128_4129DELAA p.S1377Rfs*3	F	58	Renal Breast Ovarian Sarcoma	53 breast 62 ovarian	Throat Unknown
*BRCA1*	c.68_69DELAG p.E23Vfs*17	M	0.5 (Wilm’s tumor)	Renal CNS Pheochromocytoma	–	Breast Prostate Gastric
*BRCA2*	c.7558C>T p.R2520*	F	37	Renal	–	Renal Breast CRC
*BRCA2*	c.8575C>T p.Q2859*	M	52	Renal	–	Renal Breast Pancreatic CRC Thyroid Lung
*BRCA2*	c.2731DELG p.E911Kfs*4	M	57	Renal	–	No cancers
*BRCA2*	c.425G>A p.S142N	F	51	Renal Breast	51	Breast Pancreatic Thyroid Lung Skin
*BRCA2*	c.2339C>G p.S780*	F	60	Renal Uterine	–	Renal Breast Pancreatic Prostate Sarcoma Bladder Thyroid Lymphoma
*BRCA2*	c.3847_3848DELGT p.V1283Kfs*2	M	46	Renal Prostate	53	Breast Pancreatic Prostate
*BRCA2*	c.7069_7070DELCT p.L2357Vfs*2	M	54	Renal Lung	–	Renal Breast Lung
*BRCA2*	c.518DELG p.G173Vfs*12	F	56	Renal Breast	59	Renal Breast Ovarian Prostate CRC Thyroid Lung
*BRCA2*	c.4284DUPT p.Q1429Sfs*9	M	56	Renal Pancreatic	56	Renal Breast Pancreatic Gastric
*BRCA2*	c.9294C>G p.Y3098*	F	51	Renal Breast CRC Uterine	49	Renal Prostate Brain

Patient demographics, age of diagnosis, and cancer histories are listed. Family cancer histories listed for those who reported a family history.

*CNS* central nervous system, *CRC* colorectal cancer, *HBOC syndrome* hereditary breast ovarian cancer syndrome, *RC* renal cancer, *fs* frame shift, **Ashkenazi Jewish. Family histories as self-reported on the intake form/medical records and have not been validated.

### No correlation between age of RC diagnosis and type of PV in RC

**Figure 3:**
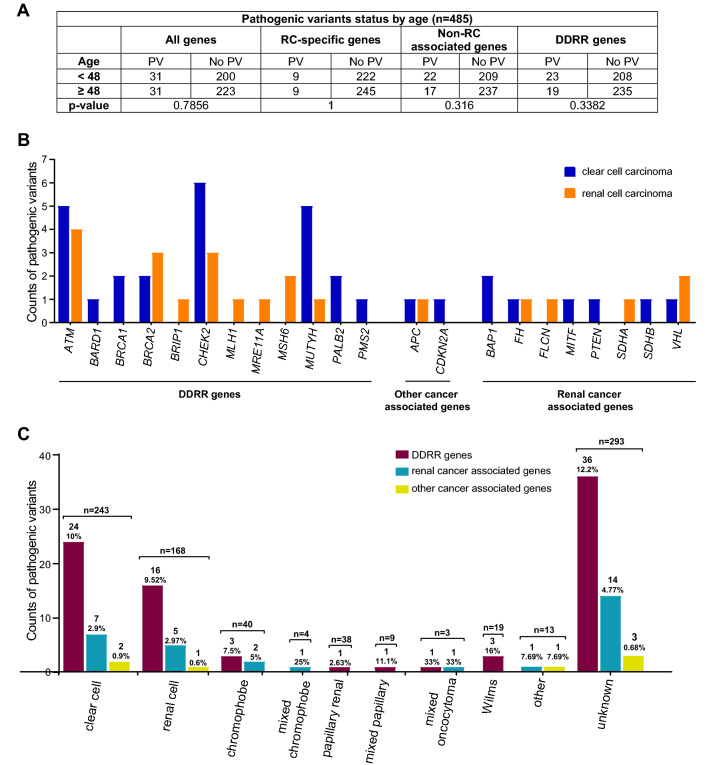
Identified PVs compared to age of RC diagnosis, and histology of RC. (**A**) Statistical comparison of PVs in all genes, RC-specific genes, non-RC and DDRR genes in younger individuals (< 48) versus older individuals (≥ 48). N = 485, these cases were tested for all 49 genes; all cases with Wilms tumor were removed. The results were non-significant (p > 0.05) using two-sided Fisher’s exact tests. (**B**) Counts of PVs by RC histology: clear cell (blue bar) and renal cell (orange bar) carcinoma in the study cohort. The' renal cell' subtype, is likely clear cell, but this cannot be confirmed. (**C**) Counts of PVs by all RC histology observed in the study cohort. DDRR genes (maroon bars), other-cancer associated genes (yellow bars), RC-specific genes (blue bars). The total number of individuals with a PV and percent PVs per gene category is shown above the bar. (**B**,**C**) Includes counts from both homozygous and heterozygous carriers of *MUTYH,* and carriers of a *FH* variant that is currently considered to be pathogenic only in the compound heterozygous or homozygous state.

To determine if identification of specific classes of germline PV correlated with age of diagnosis in this cohort, genes were divided into four broad (overlapping) categories: all genes in the panel, RC-specific genes, non-RC genes (including DDRR genes) and DDRR genes (see “Methods”). The groups were compared to median age at first RC diagnosis of < 48 or ≥ 48 years. Given the invariable early-onset of Wilms tumor, the 20 individuals with this diagnosis were excluded from the analysis. Within this eoRC cohort, there was no significant association between age at diagnosis of RC and the type of PV for any of the four broad categories above (Fig. 3A).

### Correlation of renal histologies with PVs in specific genes

Of the 243 clear cell cases in this cohort, 13.6% (33/243) had a PV, of which 2.9% were RC-associated PVs. Similar findings were observed for the cases described as renal cell carcinoma, 13.1% (22/168) had a PV, of which 2.4% were RC-associated. DDRR gene PVs were found in 24/243 (~ 10%) of clear cell cases, and in 16/168 (9.52%) of renal cell cases. Figure 3B,C contrast the findings in clear cell and renal cell histology with the other non-clear cell histologies.

## Discussion

This study for the first time demonstrates that PVs in multiple DDRR genes occur in patients with eoRC. Importantly, this study found that DDRR gene PVs were represented both in cases diagnosed with eoRC and additional cancers, and also cases diagnosed with eoRC alone. Comparison with a large control population indicated that germline PVs in DDRR genes were more common in this study cohort than in the control population, although further studies are required to confirm this finding and estimate the penetrance of PVs in DDRR genes for eoRC. We also found that germline testing using an RC-specific panel would have identified only 3.7% (18/491) of the RC cases with actionable PVs according to the NCCN recommended screening or management guidelines, compared to the 9.16% (45/491) additional cases identified with the expanded panels.

The most common gene with PVs identified in the patients in this study was the DDRR gene, *CHEK2* (19/844, 2.25%). This is consistent with recent reports by Carlo et al. and Huszno et al.^15,16^. While evidence is mounting that *CHEK2* PVs may increase risk for RC, in this study we did not consider *CHEK2* as a gene typically associated with RC as it is not currently included on RC panels and would fail to be included in testing in many cases. In addition, limitations of the previous studies and the analysis reported here together indicate that larger studies with appropriate controls are needed before confirming that *CHEK2* indeed confers a risk for RC.

Identification of germline DDRR gene PVs can have specific implications for the proband and the family. For example, 1.7% of cases diagnosed with eoRC alone had PVs in *BRCA1* or *BRCA2*, but not all of these cases had a family history strongly indicative of HBOC syndrome. This is important because identification of a *BRCA* PV can potentially change medical management; for instance, PARP inhibitor therapy is effective in tumors with *BRCA* PVs, including non-breast tumors^41,42^. Also, screening and prevention of HBOC-syndrome cancers would likely be increased significantly in the proband and in family members found to have the same PV. Further, many of the specific PVs identified in this study have been annotated as relevant to various HCPS, emphasizing their role in the development of multiple cancer types. Our results support broader panel testing as a way to identify unexpected high-penetrant PVs in eoRC patients, when there is a personal or family history of additional cancers (especially an HBOC-syndrome cancer).

In RC, a number of germline PVs have been associated with treatment response. For example, bevacizumab with everolimus or erlotinib were added as treatment options for RC cases with germline PVs in *FH*^8,43^. Currently, the clinical significance of PVs in DDRR genes is not clinically defined for RC. There is an urgent need to study the biological impact of PVs in DDRR genes in renal tissue. Such work may also lead to improved understanding of RC pathogenesis. Studies are in progress to assess cancer risk in different tissue types, and response to treatment due to a germline defect in DDRR genes^44^. A recent study showed that *VHL* inactivation in RC led to reduced expression of DDRR genes (such as *BRCA1* and *BRCA2*), and thereby increased sensitivity to PARP inhibitors^45^. These results indicate that RC tumors with DDRR gene vulnerabilities may be responsive to PARP or other DDRR gene inhibitors under development. Ongoing clinical trials are assessing the effect of PARP inhibitor, olaparib, in patients with somatic DDRR gene variants in the setting of metastatic RC (NCT03786796). Finally, it is also important to estimate the penetrance of DDRR gene PVs to clinically define RC risk. These studies will assist in genetic counseling of RC patients and their families.

The limitations of this study include the following: this is a relatively small cohort, and not all cases were tested for all 49 genes. The cohort is not representative of all individuals with RC, as the individuals reported in this study likely had clinical characteristics (e.g. high rate of additional primary cancers) or family history that led to the expanded panel testing. In the study cohort, females were overrepresented, even though more males are typically diagnosed with RC (Fig. 1B)^46^. This difference may reflect the observation that women are more likely to pursue genetic testing than men, or the fact that 34.4% of cases also had a diagnosis of breast, ovarian, or uterine cancer. Alternatively, men diagnosed with RC might be considered high-risk due to smoking or other environmental factors that lead their physician to be less suspicious of a hereditary component. A large percentage (34.7%) of tumors from the study cohort was listed as “unknown subtypes”, limiting comparison of PVs and RC histology types. Finally, matched (such as age and gender) comparisons were not possible using the large publicaly available control population (ExAC and gnomAD databases), and we made no adjustment for population stratification. Differences in the study cohort, and the large publicly available control population (ExAC and gnomAD databases) in ascertainment strategies and data collection (i.e. bioinformatic pipeline for variant calling/filtering, sequence coverage, race/ethnicities) prevent us from making any conclusions about the relationship between the PVs and RC risk^28,47–49^. The comparisons performed in this manuscript were not adjusted for multiple testing.

## Conclusions

This study is the first to indicate a role for PVs in multiple DDRR genes in eoRC. These results need to be validated in other large data sets. Additionally, to fully elucidate the biological relevance of DDRR genes to RC, family and functional studies are needed as a next step to quantify the associated risks.

## Supplementary information

Supplementary information.

## Data Availability

All data generated or analysed during this study are included in this published article [and its supplementary files].
